# Training in a novel advanced augmented reality-based ablation simulator: Using 3-dimensional magnetic resonance heart models of individual patients improves performance of future electrophysiologists

**DOI:** 10.1016/j.hroo.2026.03.020

**Published:** 2026-03-25

**Authors:** Nora Bacha, Ambra Masi, Panagiotis Antiochos, Cheryl Teres, Mathieu Le Bloa, Patrizio Pascale, Adrian Luca, Jorge Solana, Sofia Petropoulou, Samir Bengueddache, Betim Redzepi, Udaranga Wickramasinghe, Georges Caron, Hussein Ballan, Pascal Fua, Etienne Pruvot, Juerg Schwitter

**Affiliations:** 1 Faculty of Biology and Medicine, University of Lausanne, Lausanne, Switzerland; 2 Cardiology, Lausanne University Hospital, CHUV, Lausanne, Switzerland; 3 Interventional MRI Center, Lausanne University Hospital, CHUV, Lausanne, Switzerland; 4 ADIS SA – Advanced Interactive Systems, Lausanne, Switzerland; 5 Computer Science, Federal Institute of Technology Lausanne, EPFL, Lausanne, Switzerland

**Keywords:** Augmented reality simulation, Right atrial flutter ablation, Electrophysiology training, Artificial intelligence, Magnetic resonance imaging, Active tracking ablation catheters

## Abstract

**Background:**

Arrhythmias are rising worldwide owing to population aging. Efficient ablation training of next-generation electrophysiologists (EPs) is crucial. Dedicated simulators for EP training based on individual patient anatomy do not exist yet.

**Objective:**

This study aimed to assess the potential training effects of a novel advanced augmented reality (AAR)–based simulator for ablations.

**Methods:**

An AAR-based simulator (ADIS SA, Lausanne, Switzerland) tracks the movements of a physical ablation catheter (Imricor, Burnsville, MN) in real time within a completely virtual 3-dimensional heart generated from cardiac magnetic resonance examinations. Experienced EPs (n = 4) and non-EP cardiologists (n = 5) were trained in catheter steering skills. They performed 5 baseline right atrial flutter ablations (RA-FlAbls) in 5 different hearts, another 15 RA-FlAbl for training, and again 5 RA-FlAbl in the 5 baseline hearts. Efficacy (procedure time/catheter trajectory) and safety (high-force wall contacts >50 g) were recorded for coronary sinus cannulation and cavotricuspid isthmus ablations.

**Results:**

224 RA-FlAbl were performed. The training effect was evaluated using the 5 baseline and 5 post-training RA-FlAbl (43 pairs, 4 dropouts). Before training, non-EPs had 2.6 times longer procedural time and catheter trajectories than EPs (each *P* < .001; after training each nonsignificant) and 2.0 times more high-force wall contacts (*P* < .04; after training nonsignificant). EPs also demonstrated modest improvements in ablation efficacy (*P* < .003), but not for the simpler task of coronary sinus cannulation.

**Conclusion:**

This feasibility study shows that training by means of an augmented reality-based simulator improves both the efficacy and safety of catheter steering skills for RA-FlAbl of EP trainees. After the training, they obtained a comparable efficacy and safety profile as experienced EPs.

## Graphical abstract

**Figure:**
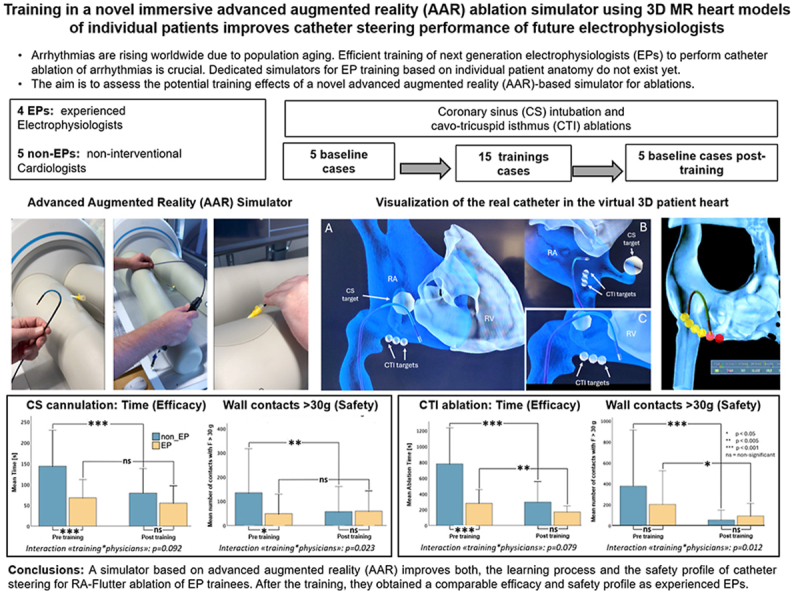

**Key Findings**

▪ Arrhythmias are rising worldwide and efficient training of next-generation electrophysiologists (EPs) to perform successful catheter ablations is crucial. To allow for training on individual heart anatomies, a novel advanced augmented reality (AAR)–based simulator was developed and tested.
▪ Cardiologists without electrophysiological formation (non-EPs) and experienced EPs were trained for right atrial flutter ablations (RA-FlAbls) using a real ablation catheter in 20 different AAR scenarios displayed on different heart anatomies. Catheter handling skills were trained but not electrogram interpretation. Non-EPs significantly improved handling skills and improved efficacy and safety of coronary sinus intubation and cavotricuspid line ablation.
▪ After training, non-EPs obtained a comparable efficacy and safety profile as experienced EPs. Experienced EPs improved slightly in terms of efficacy.
▪ The AAR simulator has the potential to accelerate trainee proficiency and enhance patient safety by enabling the acquisition of essential skills in a controlled, risk-free environment. The simulator also allows practicing rare scenarios, preparing complex individual cases, and managing potential complications without any risk to patients.

## Introduction

Electrophysiological procedures are increasing in number and complexity as the prevalence of arrhythmias is rising owing to population aging and an increase in sedentary lifestyle and metabolic syndrome.^1^^,^^2^ Consequently, efficient training of next-generation electrophysiologists (EPs) to perform catheter ablation of arrhythmias is crucial. Catheter ablation procedures require theoretical knowledge to ensure endocavitary signals comprehension and manual skills to perform ablations. Although some part of the procedure can be taught theoretically, practical training is also most important to acquire catheter steering skills to perform catheter ablation procedures safely and efficiently.

Currently, the learning process for catheter ablation procedures is based on supervised interventions in the setting of an apprentice/master model.^3^^,^^4^ Importantly, learning curves throughout the training course^5^ and higher complication rates have been reported at the beginning of the EP training.^6^^,^^7^ For this reason, simulator-based training to acquire catheter steering skills is highly desirable to shorten learning curves and increase both efficacy and safety.^8^ Moreover, training on a simulator allows to repeat interventions without exposing patients and trainees to radiographs.^8^ With these advantages, simulation can complement traditional training method by offering trainees access to “easy” and “complex” cases according to their skill profile.^9^

These advantages were acknowledged in a recent survey performed by the European Heart Rhythm Association in which 81% of EPs in Europe considered EP simulators useful for training and 96% of responders believed that simulator programs should be part of the routine institutional EP training.^10^ However, only 18% of responders had access to an EP simulator at their institution. The need of EP simulator training is also acknowledged in the most recent core curriculum for the heart rhythm specialist of the European Heart Rhythm Association^3^ and encouraged as an emerging training tool in US training statement.^4^

A variety of different simulator platforms for electrophysiological procedures exist ranging from surface and intracardiac electrocardiographic simulations primarily offering training of knowledge^11^ to more sophisticated simulators offering training for catheter steering skills.^8^^,^^9^^,^^12^^,^^13^ The latter category can be broken down into those using real catheters that are introduced into a physical heart model, typically a 3-dimensional (3D)-printed heart, or those using a “hybrid” approach where real catheters are introduced into a virtual 3D heart. In classical augmented reality (AR) systems, the real world is augmented by digital information overlaid onto the real world.^14^ In the presented system and that of others,^8^^,^^9^ the virtual world (ie, the patient’s 3D heart) is augmented by a real catheter that is “embedded” into the virtual 3D heart. In a broad sense, this is a mixed reality system. The display of real catheters is augmented by a 3D virtual heart, and the operator interacts with the 3D virtual heart by the manipulation of real catheters thereby representing a kind of high-end or advanced AR (AAR) system.

The currently available systems for training of catheter steering skills exhibit several limitations. 1 of these is the restriction of the simulation environment to a single or at least limited number of cardiovascular anatomies,^8^^,^^9^^,^^12^^,^^13^ particularly in those using a physical heart model.^12^^,^^13^ To the best of our knowledge, simulators for EP training based on individual patient anatomies do not exist yet, representing a significant unmet need in current training paradigms. To address these limitations, we developed an AAR-based simulator (AAR-sim), which is based on real patient’s magnetic resonance (MR) scans displayed in various orientations with the real ablation catheter integrated in real time into this AAR environment. This concept allows to train on different anatomies with fully virtual 3D hearts. This feasibility study aimed to assess the potential training effect on catheter steering skills of noninvasive cardiologists and experienced EP specialists when performing ablations of typical right atrial flutter ablation (RA-FlAbl) in the simulator. This arrhythmia was chosen for this study given that it is the most common reentrant arrhythmia affecting more than 300 of 100,000 person-years in people aged 50 years and older.^15^

## Methods

### Creation of RA-Fl ablation tasks for training

#### Acquisition of 3D cardiac data

For the creation of 3D heart models, the simulator requires 3D cardiac image data sets either cardiac MR based or cardiac computed tomography based. In this study, we used a 3D free-breathing electrocardiogram-triggered radial steady-state-free-precession acquisition with self-navigation for respiratory motion correction^16^ to produce the input data. The imaging parameters were as follows: repetition time/echo time 3.1/1.56 ms, field of view 182–220 mm, matrix 192, receiver bandwidth 898 Hz/pixel, isotropic spatial resolution (both acquired and reconstructed) 0.88–1.15 mm, and 12,417–15,050 radial readouts over 377–953 heartbeats. The trigger delay was set manually to acquire the data in mid-diastole. The research reported in this article adhered to the Declaration of Helsinki; the study was approved by the institutional review board of the Lausanne University Hospital, Switzerland (CER-VD, protocol 2018-00656), and all patients provided a written informed consent.

#### Creation of scenarios for RA-Fl ablations

**Figure 1:**
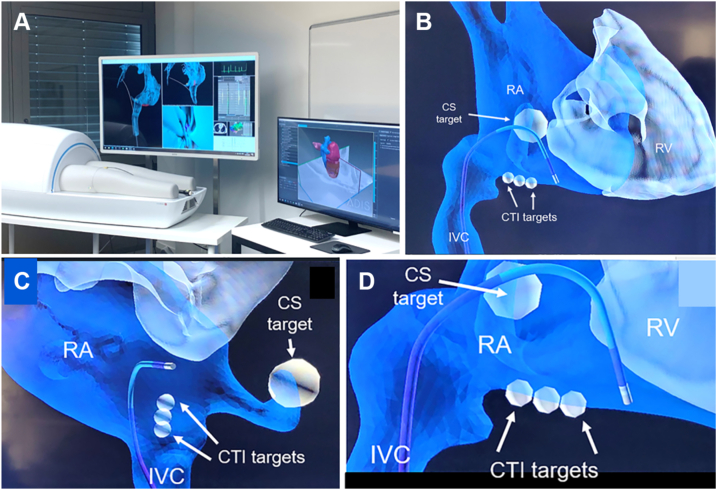
Overview of the screen during procedure. **A:** The simulator system is shown with the computer (on the right) to create the scenarios and the 3-dimensional simulator (on the left) for training. The interface allows you to load different scenarios, record the intervention, and store and export key parameters of performance. The simulator (screen on the left) shows the catheter motion in real time and key parameters such as force (g) against the wall. **B–D:** The heart is visualized in different 2-dimensional projections simultaneously. An example of a 3-dimensional model of a right atrium and a right ventricle is shown for cannulation of the CS and ablation of the CTI. The CS is marked by the *large yellow sphere* that must be reached for successful CS cannulation. The *small white spheres* are the targets placed on the CTI. Right anterior oblique view (**B**), left anterior oblique view (**C**), and left anterior oblique view with an inferior tilt (nonstandard) highlighting the CS (**D**). CS = coronary sinus; CTI = cavotricuspid isthmus; IVC = inferior vena cava; RA = right atrium; RV = right ventricle.

**Supplementary Figure 1:**
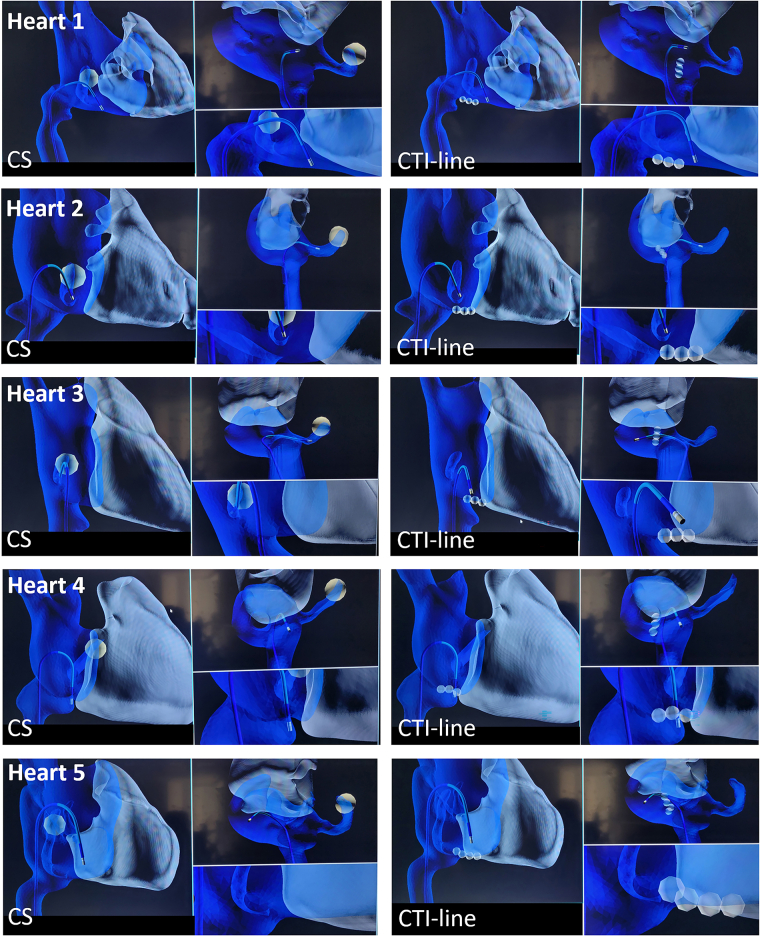


To create the various RA-FlAbl scenarios, the 3D MR imaging (MRI) data were loaded into the ARTS platform that consists of the ARTSim and the HeARTS artificial intelligence software (ADIS SA, Lausanne, Switzerland). This simulator development started in summer 2020 and is based on AR technology. Real ablation and mapping catheters are used with the simulator, and they are mapped to motion of the catheters inside virtual hearts using the proprietary technology developed by ADIS SA. This technology includes a “collision analysis,” which automatically restricts possible catheter positions to intracardiac chambers and calculates forces exerted by the catheter tip on the virtual heart walls. These forces are also displayed on the screen in real time. A first version was available for this feasibility study in fall 2023. In the first step, the 3D heart models were created representing all 4 heart chambers and the great vessels, as well as the coronary sinus (CS) as presented in Figure 1 by using the HeARTS artificial intelligence software. In the second step, various scenarios were created by adding manually 3–6 targets of 3 mm diameter on the cavotricuspid isthmus (CTI) of the 3D heart models. In each heart (n = 5), 4 slightly different CTI lines were created providing a total of 20 scenarios for RA-FlAbl (4 different CTI lines in 5 different hearts, see also Supplemental Figure 1). To evaluate the potential training effect, 5 baseline scenarios were used from 5 anatomically different hearts to produce the “baseline RA-FlAbl” data set. After training on the other 15 RA-FlAbl scenarios, the same 5 baseline scenarios were used by all physicians providing the “post-training RA-FlAbl” data set.

#### Performing the RA-FlAbl in the AAR-sim

This AAR-sim (ARTSim, ADIS SA) incorporates innovative techniques for digitizing and tracking the movements of a physical ablation catheter (MRI-compatible Vision-MR ablation catheter 1.0, Imricor, Burnsville, MN) allowing for tracking in real time, thereby enabling its precise navigation within a completely virtual 3D heart (Figure 1 and Supplemental Videos 1 and 2) generated automatically by HeARTS from patients’ MRI scans. For participation in this study, that is, for performing the RA-FlAbl, we could recruit 9 physicians at our institution, including 4 experienced EPs with 14 ± 8 years (6–25 years) of ablations experience and 5 noninvasive cardiologists (2.7 ± 2.8 years; range 6 months before to 7 years after cardiology diploma) without any previous interventional or electrophysiological experience, that is, non-EPs. To train and evaluate the catheter steering skills, the interventions on the simulator consisted of 2 steps: (1) CS cannulation and (2) placing the catheter tip onto predefined targets for ablation, as presented in Figure 1. CS cannulation was deemed successful when the catheter remained on the CS target for >3 seconds. CTI line ablation was successful when 3 conditions were met: (1) the catheter tip is located within the target of 3 mm diameter, (2) the force applied onto the target ranged between 3 and 30 g, and (3) these 2 conditions are simultaneously met for a period of >5 seconds. Participants had to reach and ablate all targets successfully within 30 min. Otherwise, the ablation was rated as nonsuccessful. This 30-minute duration was deemed realistic given that such procedures were reported to last approximately 45 minutes on average when including pre- and postablation diagnostics—testing for postablation CTI block.^17^ Of note, this study evaluated the effect of simulator training on ablation catheter steering skills and did not include training of electrophysiological knowledge such as interpretation of intracavitary electrograms, which are not (yet) integrated into the simulator environment. Therefore, pre- and postablation diagnostics and testing for postablation bidirectional CTI block was not part of the study. Accordingly, if the term “successful ablation” is used, it refers to catheter steering skills that allow of ablation on a distinct location or a distinct line of lesions.

Before the study, all physicians obtained a 30-minute instruction on the principal aspects of catheter handling given by the most experienced EP. The physicians were instructed to minimize high-force contacts onto the heart walls, that is, to consider the force on the wall, which is displayed by the AAR-sim in real time.

### Collection and analysis of the training data

For CS cannulation and CTI line ablation metrics, 4 variables were recorded to evaluate physicians’ performance. 2 parameters for procedure efficacy were obtained: (1) procedure duration (seconds) starting when entering the RA and ending when the last CTI point was ablated and (2) catheter tip trajectory (cm). For trajectory distance calculations, the sum between catheter tip positions (sampling rate of coordinates 48 ms) were calculated and summed up (starting when entering the RA and ending when the last CTI point was ablated), resulting in approximately 12,000 points per intervention on average (range 1560–34,355 points/intervention). 2 parameters were obtained for procedure safety: (1) number of catheter wall contacts with force of >30 g (force-30) and (2) with force of >50 g (force-50).

### Statistical analysis

Continuous data are given as mean ± standard deviation. Drawing upon the findings of Paetsch et al^17^ who reported approximately 12 RA-FlAbl interventions necessary to achieve competency (ie, no further reduction of procedure duration) and acknowledging that our simulator environment might not reflect the real-word situation in all aspects, we decided to expose all study participants to 20 different CTI lines for ablation (4 different lines located on 5 different hearts). To analyze the performance data, a 2-way repeated measures analysis of variance (ANOVA) was used with a within-subject factor (training: baseline vs post-training) and a between-subject factor (physicians: non-EPs vs EPs). This 2-way repeated measures ANOVA statistics was used for the 4 parameters (time, distance, force-30, and force-50) to assess the training effect of CS cannulation and CTI line ablation. To assess whether duration of ablation was different for the 5 different hearts, 1-way ANOVA testing was used for the non-EPs (at baseline and post-training) and the EPs (at baseline and post-training). To correct for non-normal distribution of the output parameters, the 2-way repeated measures ANOVA and 1-way ANOVA were performed with the results given as ranks. Statistical analyses were done using SPSS version 29.0.2.0. *P* < .05 is considered statistically significant.

## Results

**Figure 2:**
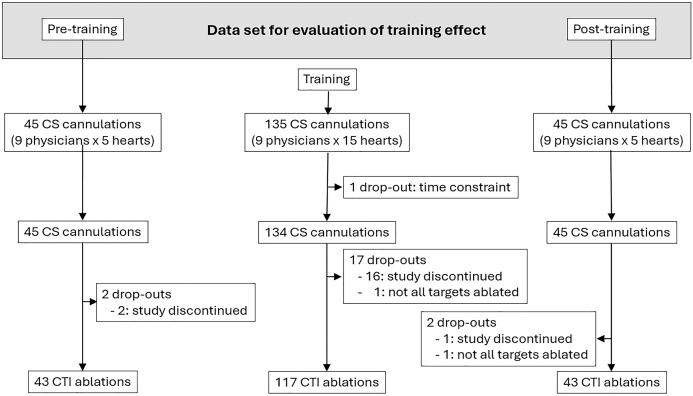
Flowchart of the study. Flowchart of all CS cannulations and CTI interventions. CS = coronary sinus; CTI = cavotricuspid isthmus.

A total of 224 of 225 planned RA-FlAbl interventions (9 physicians times 25 RA-Fl-ablations—5 at baseline, 15 for training, and 5 after training) were executed in the ARTSim. An overview of all RA-FlAbl interventions is presented in Figure 2. 1 intervention during the training phase was not executed by an EP for time constraints. Thus, 224 CS cannulations were performed successfully. Of the 224 interventions, 203 ablations were successful, that is, with all targets successfully ablated within the allotted 30 minutes. For the 21 unsuccessful interventions, 19 times the operator (EPs 11 times, non-EPs 8 times; nonsignificant by χ^2^ test) had given up before reaching the end of intervention at 30 minutes (ie, without having attempted all targets), and in 2 cases, the operator attempted all targets but had not achieved successful ablation of all targets within 30 minutes. For this study, the simulator was used on average for 3.5 hours/physician (range 1.8–7.0 hours/physician).

Regarding the baseline and post-training RA-FlAbl procedures used for the analysis of the training effect, 90 successful CS cannulation were recorded and 86 successful CTI line ablations (n = 43 for both baseline and post-training). For the 4 dropouts of the CTI line ablations, in 1 case the operator (non-EP) did not ablate all targets within the 30 minutes and 3 times (1 EP and 2 non-EPs) the operator had given up before reaching the end of study at 30 minutes.

### CS cannulation

**Figure 3:**
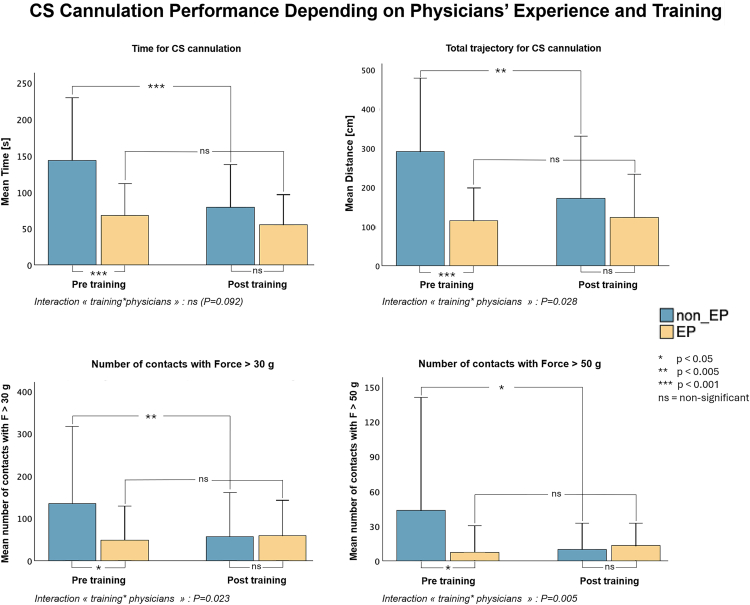
Results for CS cannulation. CS cannulation performance before and after training for EPs vs non-EPs. *Top:* Results for efficacy (time and trajectory length) for CS cannulation. *Bottom:* The safety results (number of wall contacts with force of >30 g and >50 g). At baseline, non-EPs performed significantly worse than EPs for both efficacy and safety parameters. Non-EPs improved on all parameters after training, whereas EPs have not shown significant improvement. After training, non-EPs metrics were comparable in efficacy and safety with EPs. CS = coronary sinus; EP = electrophysiologist.

At baseline, non-EPs needed 2.1 longer time than EPs to cannulate the CS (143 ± 14 seconds vs 68 ± 16 seconds, respectively; *P* < .001), as presented in Figure 3, Table 1, and Supplemental Video 1. Post-training differences were no longer present with 79 ± 10 seconds vs 55 ± 12 seconds (*P* = .095) for non-EPs vs EPs, respectively. The comparison of baseline with post-training for each group of physicians demonstrates that non-EPs benefit from training (*P* < .001), whereas EPs did not (*P* = .168 vs baseline). The training × physician interaction did not reach significance (*P* = .092).

**Table 1:** Results

Skills and safety metrics	Baseline			Post-training			Baseline to post-training		Interaction
	Non-EP	EP	*P* value (non-EP vs EP)	Non-EP	EP	*P* value (non-EP vs EP)	*P* value (non-EP)	*P* value (EP)	*P* value (training × physicians)
Ablation time (s)∗	735 ± 73	281 ± 78	<.001†	294 ± 43	171 ± 47	.054	<.001†	.003†	.079
Ablation distance (cm)∗	1086.4 ± 122.2	422.9 ± 131.5	<.001†	326.9 ± 43.8	231.5 ± 47.2	.116	<.001†	.002†	.047†
Coronary sinus cannulation time (s)‡	143 ± 14	68 ± 16	<.001†	79 ± 10	55 ± 12	.095	<.001†	.168	.092
Coronary sinus cannulation distance (cm)‡	290.8 ± 30.0	115.7 ± 33.6	<.001†	172.3 ± 27.7	124.0 ± 31.0	.258	.002†	.956	.028†
Force ablation: >30 g∗	419 ± 94	206 ± 106	.217	55 ± 21	91 ± 24	.206	<.001†	.044†	.012†
Force ablation: >50 g∗	80 ± 27	41 ± 29	.042†	7 ± 5	9 ± 5	.475	<.001†	.132	.005†
Force coronary sinus: >30 g‡	135 ± 29	48 ± 33	.015†	57 ± 19	59 ± 21	.813	.004†	.652	.023†
Force coronary sinus: >50 g‡	44 ± 15	7 ± 17	.028†	10 ± 4	13 ± 5	.404	.015†	.090	.005†

EP = electrophysiologist.

∗ Ablation of the cavotricuspid isthmus line: 43 were performed, each at baseline and post-training.

† Statistically significant.

‡ Coronary sinus cannulation: 45 were performed, each at baseline and post-training.

At baseline, the catheter tip distance for CS cannulation was 2.5 times longer for non-EPs than EPs (distance 290.8 ± 30.0 cm vs 115.7 ± 33.6 cm, respectively; *P* < .001) (Figure 3, Table 1). After training, distances for non-EPs vs EPs were no longer different (*P* = .258) with 172.3 ± 27.7 cm vs 124.0 ± 31.0 cm, respectively. As for duration, non-EPs benefit from training (*P* = .002), whereas EPs did not (*P* = .956). The training × physician interaction for catheter tip distance was significant (*P* = .028), indicating that the training effect is different for non-EPs vs EPs. For wall contact force-30 for CS cannulation, non-EPs had 2.8 times more hits than EPs at baseline (135 ± 29 vs 48 ± 33 hits for non-EPs vs EPs, respectively; *P* = .015), as presented in Figure 3 and Table 1.

After training, differences were no longer significant between both groups (57 ± 19 vs 59 ± 21 hits for non-EPs vs EPs, respectively; *P* = .813). As for the performance measures, non-EPs improved their safety profile vs baseline (*P* = .004) (Figure 3, Table 1), whereas this remained nonsignificant for EPs (*P* = .652). The training × physician interaction for force-30 was significant (*P* = .023).

For wall contacts at >50 g, non-EPs had 6.3 more hits than EPs at baseline (44 ± 15 vs 7 ± 17 hits for non-EPs vs EPs, respectively; *P* = .028). Non-EPs were no longer different from EPs after training (10 ± 4 vs 13 ± 5 hits for non-EPs vs EPs, respectively; *P* = .40). Non-EPs improved during training, whereas EPs already demonstrated low numbers of contacts at baseline (Figure 3, Table 1). The training × physician interaction for force-50 was significant (*P* = .005).

### Ablation of the CTI line

In total, 43 CTI ablations were performed both at baseline and in the post-training sessions. An example of a CTI ablation is presented in Supplemental Video 2. During training, a total of 134 CTI ablations were performed (9 physicians times 15 ablations, 1 ablation was not done for time constraints; see earlier).

**Figure 4:**
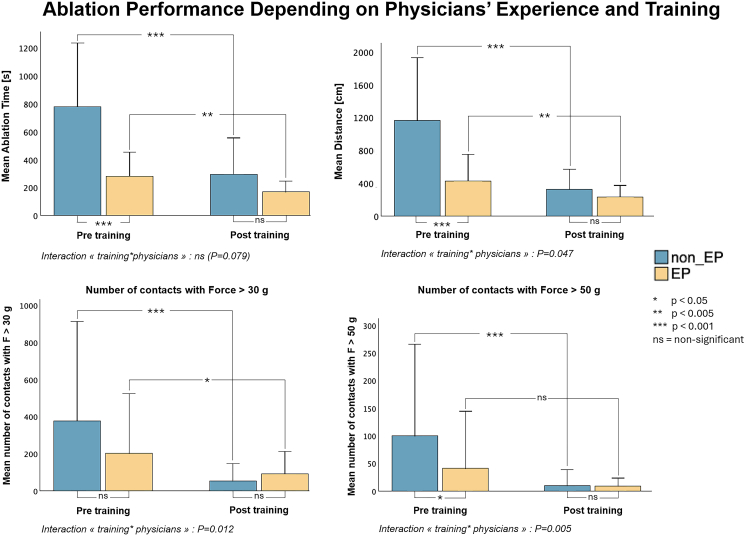
Results for catheter steering skills for CTI line ablation. Catheter steering skill performance to reach and ablate the targets on the CTI line before and after training in EPs vs non-EPs. *Top:* Results for efficacy (time and trajectory length) for ablation. *Bottom:* The safety results (number of wall contacts with force of >30 g and >50 g). At baseline, non-EPs performed significantly worse than EPs for both efficacy and the >50 g force safety parameters. Non-EPs improved on all parameters after training, whereas EPs only improved slightly in efficacy but not for force-50. After training, non-EPs were comparable in efficacy and safety with EPs. CTI = cavotricuspid isthmus; EP = electrophysiologist.

At baseline, duration of the CTI ablation procedure was 2.6 times longer for non-EPs than EPs (735 ± 73 seconds vs 281 ± 78 seconds, respectively; *P* < .001), as presented in Figure 4 and Table 1. After training, differences between the 2 groups were borderline, but no longer significant (294 ± 43 seconds vs 171 ± 47 seconds for non-EPs and EPs, respectively; *P* = .054). After the training phase, non-EPs were twice as fast compared with baseline (*P* < .001), as presented in Figure 4 and Table 1. In addition, EPs improved by a factor of 1.6 (*P* = .003) (Figure 4, Table 1). The training × physician interaction was borderline (*P* = .079). The duration of the CTI ablation procedure was not different for the 5 hearts (non-EPs at baseline *P* = .14; non-EPs after training *P* = .31; EPs at baseline *P* = .25; EPs after training *P* = .95).

**Figure 5:**
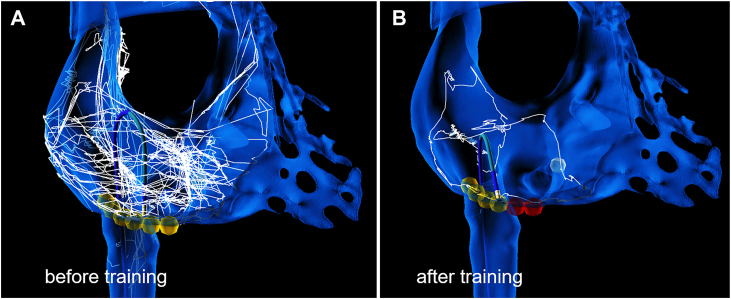
Ablation distance baseline vs post-training. An example of a catheter tip trajectory for a point on the CTI line is shown for a non-EP before (**A**) and after training (**B**). CTI = cavotricuspid isthmus; EP = electrophysiologist.

An example of catheter tip trajectory at baseline and after training in a non-EP is presented in Figure 5. Note the impressive reduction of distance indicative of a substantial improvement of catheter handling by a non-EP after the simulator training. This finding is also present for the non-EP group with a 3.3-fold reduction of catheter tip distance after training (*P* < .001) (Figure 4, Table 1). In addition, EPs improved their performance illustrated by halving the distance after training compared with baseline (*P* < .002) (Figure 4, Table 1). The training × physician interaction was significant (*P* = .047). Accordingly, there was a significant difference in distance at baseline for non-EPs vs EPs (1086.4 ± 122.2 cm vs 422.9 ± 131.5 cm, respectively; *P* < .001), which disappeared after training (*P* = .116) (Figure 4, Table 1).

Regarding the safety profile with wall contact force-30 during the CTI ablation procedure, the number of hits was similar for non-EPs vs EPs (419 ± 94 vs 206 ± 106 hits, respectively; *P* = .217). Both non-EPs and EPs improved after training (*P* < .001 and *P* < .05, respectively), and the training × physician interaction was significant (*P* = .012). For details, see also Figure 4 and Table 1.

Regarding the safety profile with wall contact force-50 during ablation at baseline, non-EPs had 2.0 times more hits than EPs (80 ± 27 vs 41 ± 29 hits, respectively; *P* = .042). Post-training differences of non-EPs vs EPs were no longer significant (7 ± 5 vs 9 ± 5 times, respectively; *P* = .475). Both groups displayed very few wall contacts at >50 g after training, with a significant improvement for non-EPs (*P* < .001) and nonsignificant one for EPs (*P* = .132). The training × physician interaction was significant (*P* = .005).

## Discussion

In this feasibility study, we aimed to evaluate the training effect of an AAR-sim to improve catheter steering skills by comparing performances of non-EPs with experienced EPs. The main findings are as follows: (1) after RA-FlAbl training on the simulator, non-EPs displayed a comparable efficacy and safety profile as experienced EPs, with a reduction in procedure duration and catheter tip trajectory (efficacy) and hits of wall contact force-30 and force-50 (safety). (2) Experienced EPs improved slightly in terms of efficacy for RA-FlAbl for the CTI line ablation compared with baseline. With high performance and safety profiles for CS cannulation at baseline, the EPs did not improve for this task. These findings highlight the simulator’s crucial role in accelerating trainee proficiency and enhancing patient safety by enabling the acquisition of essential steering skills in a controlled, risk-free environment.

### Impact of simulator training on efficacy and safety of catheter steering skills for RA-FlAbl procedures

This innovative simulator is based on a AAR platform that enables manipulation of real ablation catheters in a virtual 3D heart representation. In this AAR environment, non-EPs at baseline performed substantially worse than experienced EPs in terms of efficacy (procedure duration, catheter tip trajectory length) and safety (significant for wall contact force-50 and borderline for force-30). Thus, this simulator can differentiate various levels of skill among physicians. Most importantly, non-EPs already improved their performances after only 20 virtual ablations. Remarkably at the end of this relative short training period, non-EPs approached the skills and safety level of experienced EPs. This finding is in line with studies on fluoroscopy-based EP simulators. Studies by De Ponti et al^8^^,^^18^ showed that the use of simulation in early career EPs allowed them to improve their efficiency by improving catheter manipulation skills, reducing training time,^18^ and, thus, decreasing recurrent errors^18^ and reducing the need of help^8^ on real patients’ interventions. These studies also demonstrated that simulators could improve patient and physician’s safety by reducing fluoroscopy time.^8^ In 1 study, De Ponti et al^8^ evaluated the training effect for ablation catheter placement into the CS and onto 3 additional prespecified recording/pacing sites. These recording/pacing sites were different from our CTI line targets, but CS cannulation parameters can be used as a benchmark. The novice EP participants in the De Ponti study achieved CS cannulation after training approximately twice as fast (305 seconds at baseline vs 146 seconds after training), which is in line with our study reporting that non-EPs also cannulated the CS approximately twice as fast (143 seconds vs 79 seconds). Of note, in the study of De Ponti et al,^8^ the CS cannulation time was measured during real interventions, whereas in the current study this clinical validation is missing. Nevertheless, in the study by Paetsch et al,^17^ the CS cannulation time in patients treated by experienced EPs in the interventional MRI environment lasted 55 ± 71 seconds, which is close to the value (55 ± 12 seconds) observed in our simulator for experienced EPs after training. In our study, CS cannulation time for non-EPs before any training was 143 ± 14 seconds, which is considerably shorter than that before training in the study of De Ponti et al (305 ± 173 seconds). In addition, after training, the CS cannulation time remained substantially shorter in our study (79 ± 10 seconds) than the post-training time in the De Ponti study (146 ± 73 seconds). This difference might be attributable to the high-quality 3D visualization of the CS on MR images in our study compared with radiographic angiography images used in the De Ponti study.^8^

To the best of our knowledge, there are currently no reports available on any other AAR-sim for electrophysiological procedures. Other simulator platforms used a physical heart model to train for electrophysiological procedures.^12^^,^^13^ This physical heart–based approach was used to couple the simulator to radiation-free electroanatomic mapping systems. In 1 study, CS cannulation time by experienced EPs was 8 ± 2 seconds (vs 176 ± 89 seconds in novices).^12^ This very short cannulation time may be explained by the fact that 1 heart model was used, probably with an “easy to cannulate” CS ostium. This illustrates a limitation of a physical simulator approach, given that a whole range of full 3D printed hearts must be produced and each inserted into the simulator to expose trainees to various anatomies, whereas in the presented AAR-sim any heart anatomy from real patients can be loaded into the simulator within seconds. The presented AAR-sim also allows to train EPs according to their level of skills, train for complication management on difficult anatomies, and even train on an individual patient to prepare for specific intervention. It is also advantageous to use digitized 3D heart models in this AAR-sim, given that other tissue characteristics such as scar maps or edema maps can be merged with the 3D heart model loaded into the simulator. This will be of particular importance for the training of ventricular arrhythmia ablations. Of note, the most recent version of the simulator integrates also non-MRI-conditional ablation and mapping catheters into the 3D representation of hearts. Another advantage of the presented AAR-sim platform is given by its output, that is, quantitative metrics of performances registered continuously for discussion with the tutor. Interestingly, an improved procedural quality was achieved when the simulation training is metrics based, that is, when it is based on achieving prespecified quantitative outcomes during simulator training.^19^ We believe that the presented AAR-based training platform can offer a safe and cost-effective environment for personalized training of novice or experienced EPs to acquire and refine complex skills, respectively; to practice rare scenarios; and to manage potential complications without any risk to patients.

### Future developments

AAR-sim training was shown by De Ponti et al^18^ to improve the performance of transseptal catheterization while simultaneously reducing training time. Within a Swiss National Science Foundation–funded project the AAR-sim will be refined as a next step for transseptal catheterization and the 3D heart models will be animated by incorporating contraction and respiratory motion for a more realistic and precise visualization. As ultimate goal, the simulator is aimed for training and preprocedural planning of EP procedures of higher complexity than RA-FlAbl. To this end, intracavitary electrograms will be emulated in the next simulator version. The company (ADIS SA) plans to launch a first product simulator in the near future.

### Limitations

Several limitations need to be addressed. First, in this feasibility study, the improvement in performance as observed in the post-training interventions was not substantiated by procedures on real patients. Considering the single center setting, these first results may not be readily generalizable. To fill these gaps of evidence, further studies are needed. Nevertheless, others demonstrated that skills learned by means of an AR simulator can be transferred to actual electrophysiological^8^^,^^18^ and catheterization procedures.^20^ Second, intracavitary electrograms are not emulated in the current simulator version, which would be important for a complete realistic EP training environment. However, in the current study, we aimed to assess the impact of simulator training on the catheter manipulation skills and trainees improved substantially in both efficacy and safety parameters. Currently, the simulator is under development to integrate intracavitary electrograms that are displayed when the catheter is in contact with the virtual myocardium. Third, no haptic feedback is delivered to the operator. A safer catheter guidance could be expected, if the operator receives not only visual but also haptic feedback. Of note, at the bottom of the screen the present simulator displayed contact forces in grams of the catheter tip. In the future version, the catheter tip will be changing its color in relation to the recorded forces allowing for an intuitive manipulation by the operator. Forth, regarding the learning curve during training, a detailed analysis of all training ablation procedures was not performed owing to the extensive analyses load. Furthermore, the fact that the EPs still showed a small but significant improvement after training is indicative that the non-EPs did not yet achieve the full training effect during the study. Fifth, retention of skill, particularly in the non-EPs, was not evaluated in this feasibility study. Finally, the next version of the simulator will support the use 3D cardiac computed-tomography images as input.

## Conclusion

After RA-FlAbl training on the AAR-sim, this feasibility study shows that non-EPs obtained a comparable efficacy and safety profile for catheter steering skills as experienced EPs. Experienced EPs also improved slightly in terms of efficacy for RA-FlAbl by training on the simulator. Future studies are warranted to demonstrate translation of improved skills obtained by training in the AAR-sim into real interventions.

## Disclosures

G. Caron applied for patent. The other authors have no conflicts of interest to disclose.

## Footnotes

Appendix Supplementary data associated with this article can be found in the online version at https://doi.org/10.1016/j.hroo.2026.03.020.
