# Supplementary material for: Training in a novel advanced augmented reality-based ablation simulator: Using 3-dimensional magnetic resonance heart models of individual patients improves performance of future electrophysiologists
Source: Heart Rhythm O2. 2026 Mar 25;7(7):1314–23. doi: 10.1016/j.hroo.2026.03.020 (PMC13390052; doi:10.1016/j.hroo.2026.03.020)
Supplement: Supplementary Material [file mmc1.docx]

**Supplemental Material**

**Supplemental Figure 1:** **3D models of the 5 hearts**

The 3D models of the 5 hearts are shown in different views for coronary sinus (CS) cannulation (left column) and for reaching and ablating the cavo-tricuspid isthmus (CTI) (right column). For each heart, 4 slightly different CTI lines were defined in the simulator (by NB) to obtain 20 different ablation scenarios. For clarity, only one scenario per heart is shown. The big sphere in the CS represents the target to be reached by the trainee for successful CS cannulation. The targets on the CTI line shown as white smaller spheres must be reached and ablated by the trainee.

**Supplemental Video 1:** **Cannulation of the coronary sinus**

The video shows cannulation of the CS to reach the pre-defined CS target using the AAR simulator from a real patient anatomy.

**Supplemental Video 2:** **Ablation of the CTI line**

The video shows ablation along the CTI line, i.e. the pre-defined targets on the CTI in the AAR simulator from a real patient anatomy. The yellow targets change color to white if touched by the ablation catheter, it changes to pink if the target force is achieved (in the range of >3 g and <30 g), and red if the desired force is applied for 5 sec, which is indicative of a successful ablation. Note, that the force on the predefined target is continuously displayed on the left end of the gray bar in bold (F [g]
